# Usefulness of Xpert MTB/RIF and Xpert Ultra to Categorize Risk of Tuberculosis Transmission to Household Contacts

**DOI:** 10.1093/ofid/ofae450

**Published:** 2024-08-06

**Authors:** Alexander Mol, Adrián Sánchez-Montalvá, Juan Espinosa-Pereiro, Maria Luisa Aznar, Fernando Salvador, Pau Bosch-Nicolau, Maria-Luiza de Souza-Galvão, María Ángeles Jiménez, José Ángel Rodrigo-Pendás, Joan-Pau Millet, Nuria Saborit, Claudia Broto, Israel Molina, Teresa Tórtola

**Affiliations:** International Health Unit Vall D’Hebron-Drassanes, Infectious Diseases Department, Vall d’Hebron University Hospital, PROSICS Barcelona, Barcelona, Spain; International Health Unit Vall D’Hebron-Drassanes, Infectious Diseases Department, Vall d’Hebron University Hospital, PROSICS Barcelona, Barcelona, Spain; Grupo de Estudio de Infecciones por Micobacterias, Sociedad Española de Enfermedades Infecciosas y Microbiología Clínica, Madrid, Spain; Center for Biomedical Research in Infectious Diseases Network, Instituto de Salud Carlos III, Madrid, Spain; Department of Medicine, Universitat Autònoma de Barcelona, Barcelona, Spain; International Health Unit Vall D’Hebron-Drassanes, Infectious Diseases Department, Vall d’Hebron University Hospital, PROSICS Barcelona, Barcelona, Spain; Grupo de Estudio de Infecciones por Micobacterias, Sociedad Española de Enfermedades Infecciosas y Microbiología Clínica, Madrid, Spain; Department of Medicine, Universitat Autònoma de Barcelona, Barcelona, Spain; International Health Unit Vall D’Hebron-Drassanes, Infectious Diseases Department, Vall d’Hebron University Hospital, PROSICS Barcelona, Barcelona, Spain; Center for Biomedical Research in Infectious Diseases Network, Instituto de Salud Carlos III, Madrid, Spain; Department of Medicine, Universitat Autònoma de Barcelona, Barcelona, Spain; International Health Unit Vall D’Hebron-Drassanes, Infectious Diseases Department, Vall d’Hebron University Hospital, PROSICS Barcelona, Barcelona, Spain; Center for Biomedical Research in Infectious Diseases Network, Instituto de Salud Carlos III, Madrid, Spain; Department of Medicine, Universitat Autònoma de Barcelona, Barcelona, Spain; International Health Unit Vall D’Hebron-Drassanes, Infectious Diseases Department, Vall d’Hebron University Hospital, PROSICS Barcelona, Barcelona, Spain; Center for Biomedical Research in Infectious Diseases Network, Instituto de Salud Carlos III, Madrid, Spain; Department of Medicine, Universitat Autònoma de Barcelona, Barcelona, Spain; Pulmonology Department, Vall d’Hebron University Hospital, Barcelona, Spain; Pulmonology Department, Vall d’Hebron University Hospital, Barcelona, Spain; Preventive Medicine and Epidemiology Department, Vall d’Hebron University Hospital, Barcelona, Spain; Epidemiology Service, Agència de Salut Pública de Barcelona, Barcelona, Spain; Center for Biomedical Research in Epidemiology and Public Health Network, Instituto de Salud Carlos III, Madrid, Spain; International Health Unit Vall D’Hebron-Drassanes, Infectious Diseases Department, Vall d’Hebron University Hospital, PROSICS Barcelona, Barcelona, Spain; International Health Unit Vall D’Hebron-Drassanes, Infectious Diseases Department, Vall d’Hebron University Hospital, PROSICS Barcelona, Barcelona, Spain; International Health Unit Vall D’Hebron-Drassanes, Infectious Diseases Department, Vall d’Hebron University Hospital, PROSICS Barcelona, Barcelona, Spain; Center for Biomedical Research in Infectious Diseases Network, Instituto de Salud Carlos III, Madrid, Spain; Department of Medicine, Universitat Autònoma de Barcelona, Barcelona, Spain; Microbiology Department, Vall d’Hebron University Hospital, Universitat Autònoma de Barcelona, Barcelona, Spain

**Keywords:** contact investigation, PCR, prevention, risk of transmission, tuberculosis

## Abstract

**Background:**

People with pulmonary tuberculosis (PTB) are contagious, particularly to their household contacts. Their infectivity has been associated with the bacterial load in sputum samples. This study investigated if the bacterial load in sputum samples as quantified by Xpert MTB/RIF and Xpert Ultra is correlated with the extent that latent tuberculosis infection (LTBI) occurred in household contacts of people with PTB.

**Methods:**

A retrospective study was performed including people with PTB presenting at Vall d’Hebron University Hospital, Barcelona, between 2011 and 2021. Their infection ratio, representing the proportion of household members found with LTBI in contact tracing investigation, was compared with the quantitative results of Xpert MTB/RIF and Xpert Ultra using ordinal regression analysis.

**Results:**

A total of 107 people with PTB were included. Among their 398 household contacts, 126 (31.7%) cases of LTBI and 14 cases with active TB disease (3.5%) were reported. Higher bacterial load in Xpert MTB/RIF and Xpert Ultra baseline sputum was significantly associated with increased infection ratios, providing better estimates than conventional acid-fast bacilli (AFB) smear grading.

**Conclusions:**

Xpert MTB/RIF and Xpert Ultra could serve as an alternative to AFB sputum-smear grading in determining contact tracing priorities.

Despite the ambitious World Health Organization (WHO) goal of reducing 95% of tuberculosis (TB)–related deaths by 2035, TB was diagnosed in an estimated 10.6 million people in 2022 and in the same year has caused 1.3 million deaths [1]. To achieve this target, early diagnosis is important. The standard of TB diagnosis has been acid-fast bacilli (AFB) sputum-smear microscopy as well as liquid culture. In recent years, we have moved to a molecular diagnostic approach where low-complexity nucleic acid amplification tests (NAATs) are used. One of them, the Xpert MTB/RIF assay (Cepheid, Sunnyvale, California) is recommended as the initial diagnostic test for TB [2]. It uses real-time polymerase chain reaction (PCR) to detect *Mycobacterium tuberculosis* and resistance to rifampicin simultaneously [3]. Sensitivity and specificity are significantly higher than those of smear microscopy [3, 4]. Its successive version, Xpert MTB/RIF Ultra (Cepheid) (hereafter called Xpert Ultra), was developed in 2017. It has higher accuracy and a better limit of detection than previous Xpert MTB/RIF versions [5, 6].

In TB diagnostics, it is relevant to not only affirm bacterial presence but also to quantify the bacterial load, which is useful for assessment of disease severity [7] or as a proxy of infectivity [8–10]. Quantification of the bacterial load in a sample can be done by counting the number of bacteria visually under a microscope, as is done with AFB sputum-smear microscopy or using the cycle threshold (Ct) value of detection provided by real-time PCR techniques such as Xpert MTB/RIF. Xpert MTB/RIF and Xpert Ultra automatically categorize the samples in 4 categories according to bacterial load (high, medium, low, and very low) [11]. The Ct value has a log-linear relationship with the bacterial concentration in spiked sputum samples [10].

Close contacts of a person with pulmonary TB (PTB) are at high risk of developing a latent TB infection (LTBI), which may later progress to active TB disease [12]. Risk of transmission depends, among other factors, on the intensity of exposure or contact susceptibility [13]. Household contacts of people with TB are at increased risk as they are generally in close contact [14]. For low- and middle-income countries, the WHO recommends to perform contact investigation among household contacts of people with TB who are sputum-positive [15, 16]. However, it has been acknowledged that transmission still occurs in sputum-negative cases [16]. Although Xpert MTB/RIF and Xpert Ultra can detect positive cases among people with TB who are sputum negative, which might be a potential pool of transmitters, there is very scarce evidence supporting the quantification by Xpert MTB/RIF and Xpert Ultra as a driver of contact investigation.

The aim of our study was to investigate the relationship between Xpert MTB/RIF and Xpert Ultra and degree of transmission of TB to household contacts. Secondary objectives were to compare the quantification of Xpert MTB/RIF and Xpert Ultra with quantification by AFB smear grading and to examine the presence of pulmonary cavities in relationship to bacterial load by Xpert MTB/RIF and Xpert Ultra and infection ratio.

## MATERIALS AND METHODS

### Study Design

This retrospective study was performed at Vall d’Hebron University Hospital (VHUH) in Barcelona, Spain. People aged ≥16 years who were diagnosed with PTB using Xpert MTB/RIF or Xpert Ultra on a sputum sample between January 2011 and January 2021 were included. In the first years of this period, Xpert MTB/RIF was only selectively used for patients with a risk of rifampicin resistance or a high probability of false-negative AFB smear microscopy, which means in this period the majority of people was diagnosed with other diagnostic means. Sample types other than sputum samples were not useful for the purpose of this study and not included. As this study assessed the occurrence of LTBI among household contacts, people without contact tracing investigation or without household members were excluded for analysis of the primary objective.

Personal characteristics of people with TB were extracted from the electronic health management system from VHUH. Xpert MTB/RIF and Xpert Ultra results were extracted from the Xpert Cepheid server. Data of AFB smear microscopy and culture results were retrieved from the Microbiology Department of VHUH. Where data on Xpert MTB/RIF, Xpert Ultra, contact tracing investigation, or AFB smear grading were unretrievable, these people were excluded from analysis. No imputations were made on missing data of any variable.

### Microbiology

After decontamination of the samples, AFB smear microscopy and culture were performed. All samples with a positive auramine staining were stained with Ziehl-Neelsen. Sputum-smear grading was microscopically determined according to Centers for Disease Control and Prevention guidelines [17]. Mycobacterial culture was done using BD BACTEC MGIT mycobacterial growth indicator tubes (Becton Dickinson, Heidelberg, Germany).

Initially, PCR was only selectively requested for patients with a risk of rifampicin resistance or a high probability of false-negative AFB smear microscopy. Later, Xpert MTB/RIF was performed in all people with presumed TB, and in 2017 this was replaced by Xpert Ultra. When positive, the Xpert MTB/RIF and Xpert Ultra provided a semi-quantitative result, which is subsequently grouped as very low, low, medium, or high. These are calibrated by the manufacturer in such a way that these groups represent a similar bacterial load. For Xpert MTB/RIF: very low (Ct >28), low (Ct 22–28), medium (Ct 16–22), or high (Ct ≤16); and for Xpert Ultra: very low (Ct >29), low (Ct 25–28), medium (Ct 19–24), or high (Ct 15–18.9).

In case a person had multiple sputum samples, the samples that were tested both by AFB smear grading and PCR were selected. The sample with the highest AFB smear grading was selected subsequently. For quantitative analysis involving continuous Ct values, the probe that first showed positive (Ct_min_) was selected, as is done by the GeneXpert instrument in automatic quantification.

### Contact Tracing

Data on contact investigation were retrieved from the Preventive Medicine and Epidemiology department at VHUH and from Agència de Salut Pública de Barcelona, which coordinates TB activities in the region of Barcelona. It was intended to screen household contacts within 14–30 days after identification of the index case. Contacts were screened using tuberculin skin test (TST) or the QuantiFERON-TB Gold interferon-gamma release assay (IGRA), according to manufacturer's instructions. A contact was considered a case of LTBI if the TST skin lesion exceeded 5 mm in cross-section after first screening. If the first TST was <5 mm, it was repeated after 8–12 weeks. If the skin lesion in this second screening round was >5 mm, it was considered a case of LTBI as well. In BCG-vaccinated participants, the threshold was set at 10 mm for positivity. Also a household contact with a positive IGRA result was considered a case of LTBI. BCG-vaccinated contacts were preferably tested with IGRA [18].

The TB infection ratio was defined as the proportion of household contacts with LTBI or TB disease of the total number of household contacts of the TB index case. To rank and subsequently compare infection ratios, these were grouped in 4 custom-made categories: no transmission and infection ratios of 1%–33%, 34%–67%, or 68%–100%.

### Statistical Analysis

Data were described by mean with 95% confidence interval (CI) (normally distributed) or by median with interquartile range (IQR) (nonnormally distributed). Comparison of the size of the infection ratio groups among the different Xpert MTB/RIF and Xpert Ultra quantification groups was done using a contingency table assessed by the χ^2^ test, and by ordinal regression analysis. Odds ratios expressed the likelihood of having a higher infectivity ratio as a result of higher quantities of bacteria by Xpert MTB/RIF and Xpert Ultra quantification results. Ordinal regression analysis was done comparing Xpert MTB/RIF and Xpert Ultra quantification with AFB smear grading. Differences in mean Ct values provided by Xpert MTB/RIF and Xpert Ultra among the AFB smear grading groups were analyzed with 1-way analysis of variance (ANOVA). This was done side-by-side because the 2 cartridges are differently calibrated, which means the Ct_min_ values do not represent the same bacterial load. Tests were considered significant when the 2-tailed *P* value was <.05. Data were analyzed with IBM SPSS Statistics software (version 21.0.0.0; IBM SPSS, Armonk, New York).

### Patient Consent Statement

This study was approved by the Research and Ethical Committee (EC) of VHUH (ref.: PR(AG)195/2022). An informed consent waiver was granted, given the retrospective nature of the study. All personal data were processed in accordance with European regulations on the protection of personal data and the Declaration of Helsinki. The study database is available under request and upon approval by the local EC.

## RESULTS

### Characteristics of Included People

**Figure 1.**
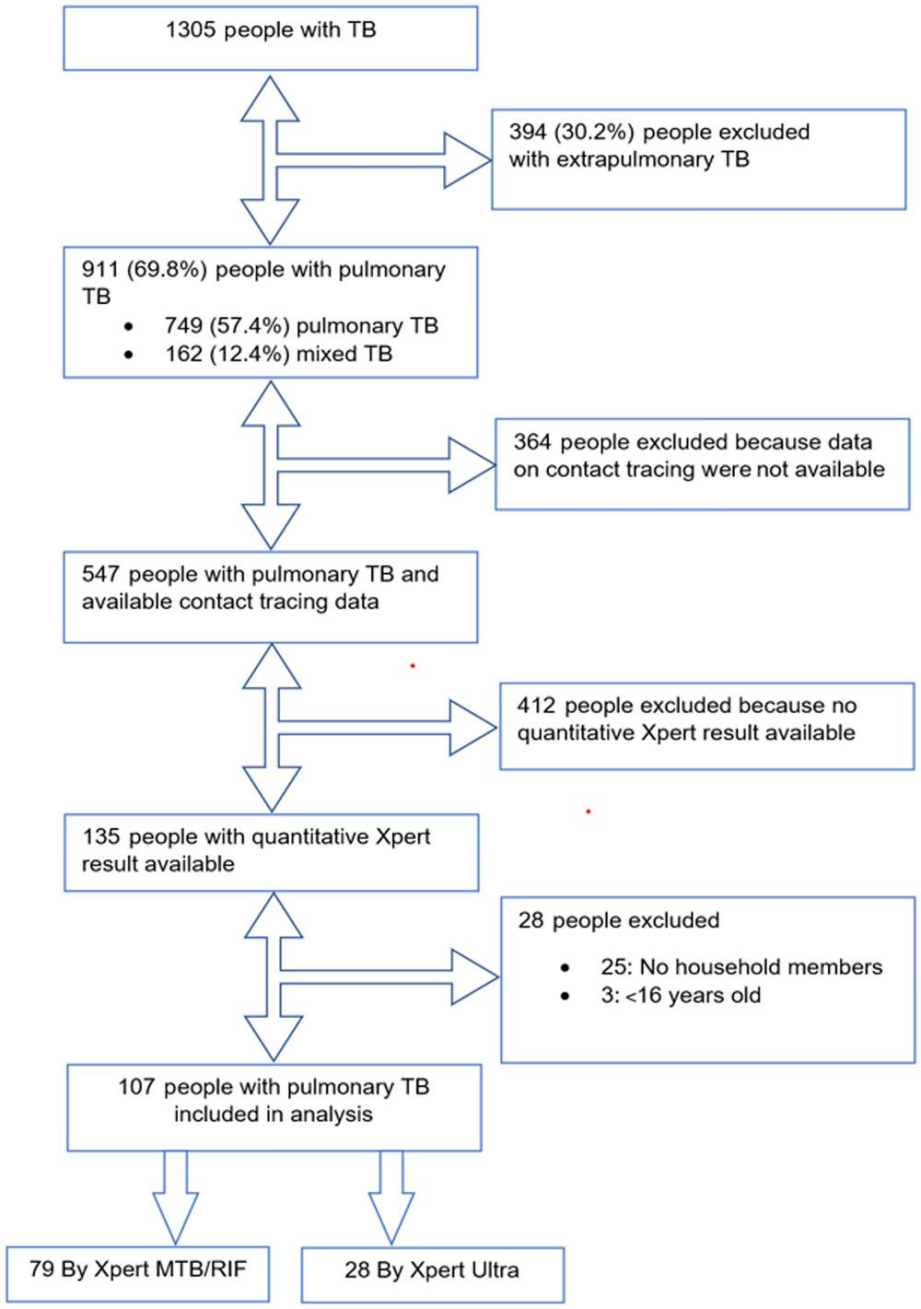
Flowchart of people with tuberculosis (TB).

A total of 1305 people with TB were identified between 2011 and 2021. Of them 542 (41.5%) were female. Mean age was 47.6 years and 841 (64.5%) were born outside Spain. In total, 749 people (57.4%) had PTB, 394 (30.2%) extrapulmonary TB, and 162 (12.4%) a combination of both. A flowchart of inclusion is shown in Figure 1. Of the 911 people with TB with pulmonary involvement, 255 (28.0%) had a positive AFB smear result and 319 (35.0%) had a negative AFB smear result in baseline sputum. Of the remaining 337 (37%) AFB smear grading had been done on other sample media than sputum or the AFB smear result was unavailable. Among people with PTB, 719 (78.9%) had a positive culture and 98 (10.8%) had a negative culture. Of the remaining 94 (10.3%), the result of culture was retrospectively irretrievable. We retrieved contact tracing data of 547 (60.0%) people with PTB. Among the subset of people with PTB with contact tracing information, we had Xpert MTB/RIF or Xpert Ultra results of 135 people. Three persons were excluded because they were younger than 16 years and 25 people because they had no household contacts. A total of 107 people were included, of which 79 were tested by Xpert MTB/RIF and 28 by Xpert Ultra. Table 1 describes their general characteristics.

**Table 1.** Baseline Characteristics of People With Pulmonary Tuberculosis

Characteristics	Cases (N = 107)
Age, y, mean (SD)	47.8 (19.3)
Sex	
Male	68 (63.6)
Female	39 (36.4)
Country of birth	
Spain	39 (36.4)
Outside Spain	68 (63.6)
BCG vaccination	17 (15.9)
Immunosuppression	
Type 2 DM	7 (6.5)
HIV	20 (18.7)
Drug induced	3 (2.8)
Autoimmune disease	1 (0.9)
Transplantation	1 (0.9)
Symptoms	
Respiratory	87 (81.3)
Weight loss	56 (52.3)
Sweating	39 (36.4)
Asthenia	43 (40.2)
Other	24 (22.4)
Radiology	
Normal	1 (0.9)
Abnormal with cavities	39 (36.4)
Abnormal without cavities	67 (62.6)
Tobacco use	
Active smoker	39 (36.4)
Former smoker	7 (6.5)
Non-smoker	61 (57.0)
AFB smear grading	
0	12 (11.2)
1	14 (13.1)
2	13 (12.1)
3	39 (36.4)
4	29 (27.1)
Xpert MTB/RIF quantification group	
Absent	7 (6.5)
Very low	6 (5.6)
Low	21 (19.6)
Medium	38 (35.5)
High	35 (32.7)
Infection ratio groups	
No transmission	39 (36.4)
1%–33%	10 (9.3)
34%–67%	28 (26.2)
68%–100%	30 (28.0)

Data are presented as No. (%) unless otherwise indicated.

Abbreviations: AFB, acid-fast bacilli; DM, diabetes mellitus; HIV, human immunodeficiency virus; SD, standard deviation.

No differences were observed between included and excluded people with PTB regarding sex, age, country of birth, previous TB, immunosuppression, or BCG vaccination (Supplementary Table 1).

### Contact Characteristics

The 107 included people listed 1165 contacts, of which 398 (34.2%) were household contacts. LTBI was found in 158 (39.7%) household contacts. Another 14 (3.5%) household contacts were diagnosed with active TB disease, in 8 different households. With a mean age of 14.1 (standard deviation [SD], 9.9 years), household members who were diagnosed with active TB disease were significantly younger than noninfected household members (mean age, 34.8 [SD, 18.7] years) or household members with LTBI (mean age, 40.2 [SD, 16.7] years) (*P* < .001; Table 2).

**Table 2.** Comparing General Characteristics of Household Members: Not Infected, Latent Tuberculosis Infection, and Active Tuberculosis

Characteristic	Overall Household Population (n = 398)	Non-infected (n = 226)	LTBI (n = 158)	Active TB (n = 14)	*P* Value
Age, y, mean (SD)	35.9 (18.4)	34.3 (18.7)	40.2 (16.7)	14.1 (9.9)	<.001^a^
Sex					
Male	241 (60.6)	134 (59.3)	96 (60.8)	11 (78.6)	.358
Female	157 (39.4)	92 (40.7)	62 (39.2)	3 (21.4)	
BCG vaccination					
No	149 (37.4)	92 (61.7)	49 (40.5)	8 (57.1)	.002^a^
Yes	135 (33.9)	57 (38.3)	72 (59.5)	6 (42.9)	
Unknown	114 (28.6)				
Country of origin					
Spain	159 (39.9)	109 (48.2)	43 (27.2)	7 (50.0)	.158
Outside Spain	239 (60.1)	117 (51.8)	115 (72.8)	7 (50.0)	

Data are presented as No. (%) unless otherwise indicated.

Abbreviations: LTBI, latent tuberculosis infection; SD, standard deviation; TB, tuberculosis.

^a^Significant difference, *P* < .05.

### Infection Ratios

**Figure 2.**
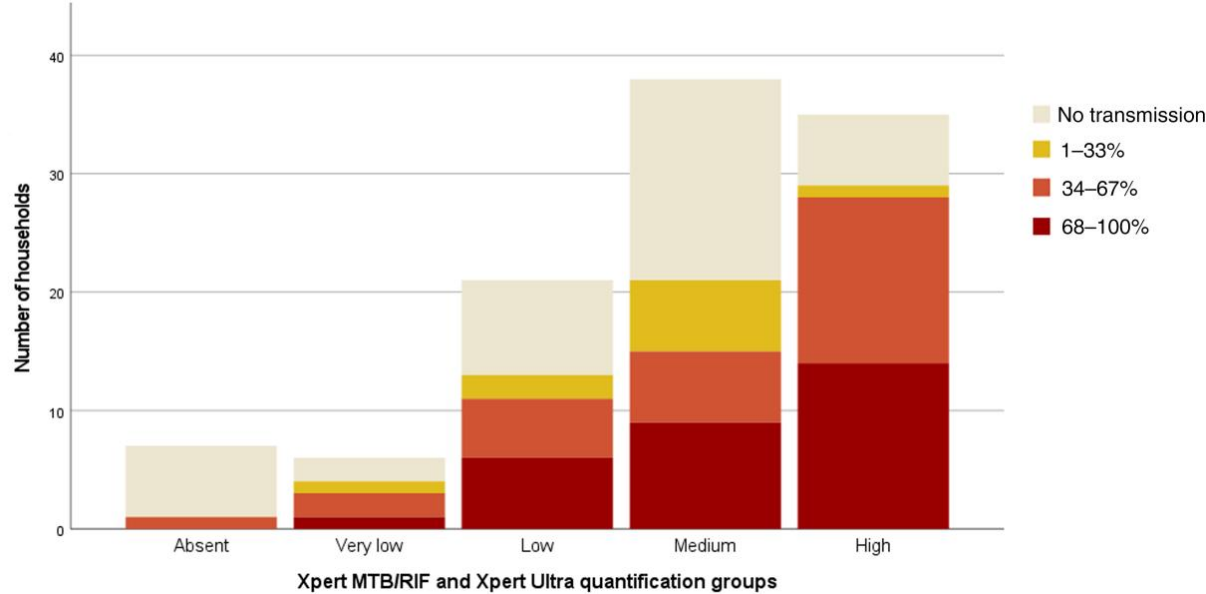
Number of households in each of the transmission categories in the household of tuberculosis (TB) index cases according to the Xpert quantification groups. The Xpert MTB/RIF and Xpert Ultra quantification groups are automatically provided by the GeneXpert system.

Included people with TB had a median household of 2 persons (IQR 2-4). One TB index case had a household of 65 people, as he was contained in a youth detention center. This person was included as it did not change sensitivity analysis. Transmission of TB was observed in 68 households (63.6%). The distribution of the grouped infection ratios according to Xpert MTB/RIF and Xpert Ultra quantification groups is provided in Figure 2. There were significantly more people with a high infection ratio in the higher Xpert MTB/RIF and Xpert Ultra quantification groups (χ^2^ = 22.2; *P* = .035; Supplementary Table 2 shows the contingency table). There was 1 person (1/7 [14.3%]) with negative Xpert MTB/RIF and a related LTBI case. For negative AFB smear grading, transmission in 4 of the 12 people (33.3%) was still observed. Regarding the household contacts found with active TB, 8 of 14 were contacts of an index case with the highest Xpert quantification group.

### Ordinal Regression

Among the same group of people with PTB, we performed a regression analysis on the predictive capacity of the Xpert MTB/RIF and Xpert Ultra quantification groups on the infection ratios, and compared this to the predictive capacity of AFB smear grading. Overall, the Xpert MTB/RIF and Xpert Ultra quantification is a positive predictor of infectivity. Moving up 1 Xpert MTB/RIF and Xpert Ultra quantification group was associated with an increase in the odds of having a higher infection ratio of 1.68 (95% CI, 1.21–2.34; Wald χ^2^ = 9.372; *P* = .002). The relationship remained constant after adjusting for the possible confounding number of household members. AFB smear grading has a similar outcome. Moving up 1 AFB smear grading category was associated with an increase in the odds of having a higher infection ratio of 1.55 (95% CI, 1.17–2.06; Wald χ^2^ = 9.402; *P* = .002).

### Mean Ct_min_ Value and AFB Smear Grading

**Figure 3.**
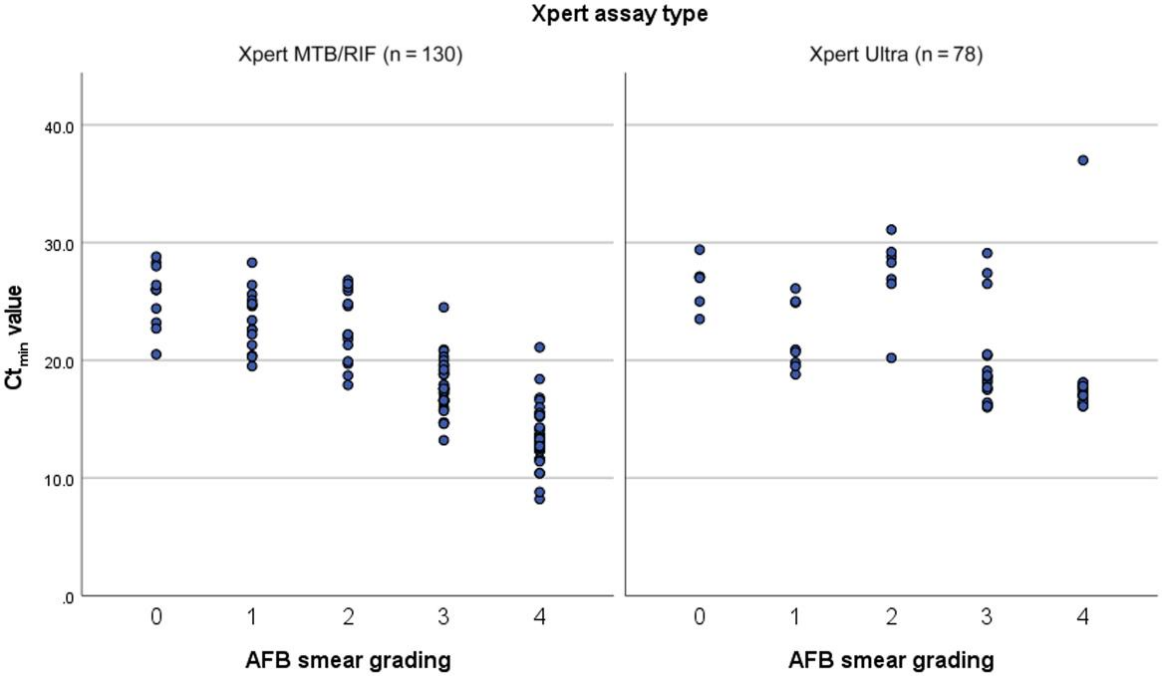
Distribution of Ct_min_ (minimal cycle threshold of the first positive probe values) for Xpert MTB/RIF (left) and Xpert Ultra (right) per acid-fast bacilli (AFB) smear grading categories. A higher AFB smear grading group represents a higher bacterial load.

We assessed the correlation between the Xpert MTB/RIF and Xpert Ultra Ct_min_ value and AFB smear grading groups using a patient subset composed of all people with PTB of whom we had data on both these variables. This was the case in 208 people, of which 130 were tested by Xpert MTB/RIF and 78 by Xpert Ultra (Figure 3). The mean Ct_min_ (95% CI) by Xpert MTB/RIF was lower in the higher AFB smear categories: group 0, 25.5 (23.8–27.2); group 1, 23.5 (22.1–24.8); group 2, 22.7 (20.9–24.5); group 3, 17.8 (17.0–18.5); group 4, 13.6 (12.8–14.4) (ANOVA Welch: *P* < .001). The mean Ct_min_ values (95% CI) by Xpert Ultra per AFB smear categories were as follows (95% CI): group 0, 26.5 (24.4–28.6); group 1, 22 (19.5–24.4); group 2, 27.3 (24.1–30.5); group 3, 19.4 (17.5–21.3); group 4, 18.2 (15.9–20.6) (ANOVA Welch: *P* < .001).

### Pulmonary Cavities

**Figure 4.**
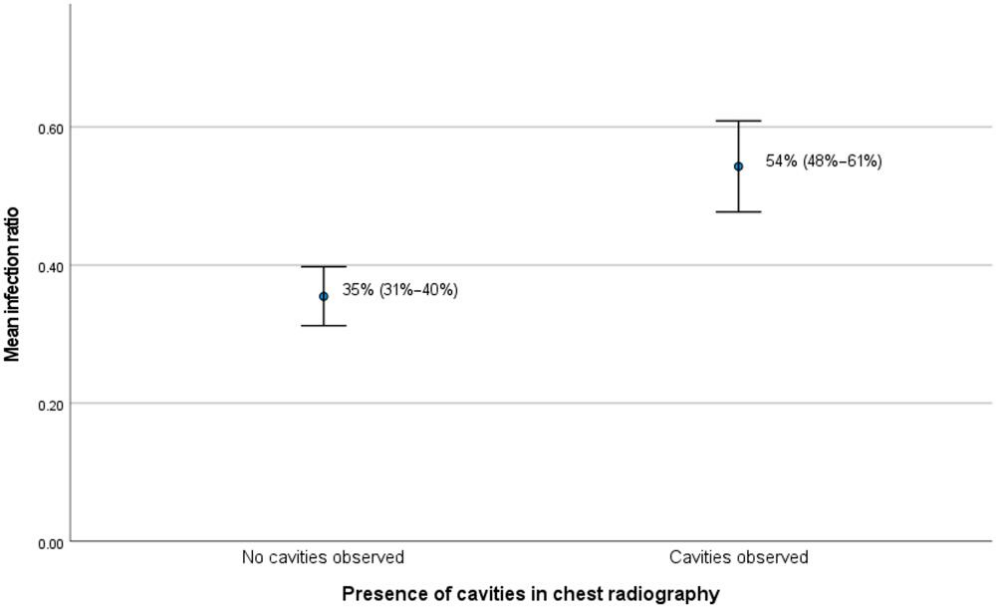
Mean infection ratio in the presence of cavities. This graph compares the mean percentage of the household contacts of people with pulmonary tuberculosis (TB) with and without lung cavities that are found with either latent TB infection or active TB (*P* = .018). Error bars indicate the 95% confidence interval.

We compared the presence of cavities with the infection ratio. We used a subset of 456 participants with information regarding radiology results and data on contact investigation available. In total, 130 people had cavities, compared to 326 people without cavities. Respectively, 99 of 130 (76.2%) and 157 of 326 (53.7%) transmitted TB to their household (*P* < .001). People with pulmonary cavities had a higher mean infection ratio than people without cavities (54% [95% CI, 48%–61%] vs 35% [95% CI, 31%–40%]; *P* = .018; Figure 4). To assess the correlation between cavities and Ct values of the molecular test, for this analysis we used participants with results on molecular test and X-ray, which was the case for 208 people. In total, 76 of 208 (36.5%) participants had cavities and 132 of 208 (63.5%) did not have cavities. People with pulmonary cavities had significant lower Ct_min_ values than people without cavities (16.2 [95% CI, 14.9–17.4] vs 19.0 [95% CI, 17.5–20.4]; *P* < .005). However, there was no correlation between presence of pulmonary cavities and the Xpert MTB/RIF and Xpert Ultra quantification groups (*P* = .053), nor with AFB smear grade quantification groups (*P* = .290).

## DISCUSSION

The results showed that household contacts of people with TB with a high bacterial load as detected by Xpert MTB/RIF or Xpert Ultra in baseline sputum are more at risk to be infected. Nowadays, contact tracing investigation is driven by the quantification of bacteria using AFB smear grading [9, 15]. Although correlation between Xpert MTB/RIF and Xpert Ultra and AFB smear grading is robust [8, 19], there is only 1 recent study, which observed that people with LTBI were more likely to be a close contact of a TB index case with lower Ct values [20]. Our result shows that the Xpert MTB/RIF and Xpert Ultra quantification can support AFB smear grading to identify people with a higher risk of TB transmission. Of special notice is that people with negative Xpert MTB/RIF or Xpert Ultra retain a lower grade of infection with respect to negative AFB smear grading. It confirms other studies showing Xpert MTB/RIF and Xpert Ultra to be more accurate than AFB smear in assessing infectivity of people with TB, as people who are AFB smear negative might still be infective [8, 16]. Moreover, the presence of cavities in TB index cases is shown to correlate with increased TB transmission to household contacts, supporting the results of earlier observations [20].

In settings with limited resources, Xpert MTB/RIF and Xpert Ultra can prioritize contact tracing, although as an alternative for sputum sampling, studies have suggested exhaled air–based sampling to predict infectivity [21–23]. Nonetheless, contact investigation should not only rely on bacterial quantification, as there is great variability in TB transmission among individuals and higher transmission rates might also be explained by differences in intrinsic bacterial factors [24, 25]. It should rather elaborate a risk probability of each household individual based on characteristics of the index case, attributes of the contact event, and the individual infection risk and progression of disease of each contact, since people with low bacteria count with long diagnostic delay may transmit for prolonged periods [26]. Other drivers such as housing conditions, overcrowding, and contacts’ susceptibility should be considered when deciding allocation of resources [15].

The low age of household contacts with active TB disease is in accordance with the higher susceptibility of the pediatric population to progress to active TB [27]. The increased infection risk might result from increased intimacy between adult and child. In addition, their immune system is still developing, which complicates controlling *M tuberculosis* expansion. Moreover, children are at higher risk of developing severe manifestations of TB disease and have higher mortality rates [28, 29].

Our study shows similar prevalence of LTBI in household contacts of people with PTB in the Barcelona region as observed in a recent study, around 35%–40% [30]. Despite the lack of external validation cohorts, we consider that our PTB participants do not differ in regard to demographic and clinical characteristics as those from other countries with low TB incidence. The study cohort is concordant with the conception of young men presenting with classical symptoms and at least one-third of them with cavities in the thoracic X-ray.

The study has several limitations. First, many people had to be excluded due to incomplete data, but no differences were found between the included and excluded populations. Unavailable outcomes of AFB smear grading, Xpert, or culture results were the most common reasons for exclusion. Second, sample size limitation affects the power of the study and prevents having narrower CIs. Third, we may face some selection bias since we found only a few people with negative Xpert MTB/RIF or Xpert Ultra results due to the fact NAAT was initially not applied systematically to all people presenting with PTB presumptive signs or symptoms. Moreover, our study recorded the status of infection of the household contact, although it is impossible to know whether the infection was recently acquired as a result of current contact or in a previous episode outside of the household environment. Adjustment by underlying risk factors of household members could not be performed due to missing medical health records. Although age and immunosuppression are more relevant risk factors of progression from latent TB to active TB, diabetes mellitus and smoking have also been related with increased risk [12]. However, we think the effect of our findings is robust, as a correlation with other infectivity proxies is strong. Treating close contacts of people with PTB is known to be a very efficacious tool to prevent TB amplification in the community. Identifying contacts with the highest risk is important to allocate resources to minimize dropouts during the process [31].

## CONCLUSIONS

This study shows a good correlation between infectivity of people with PTB among their household contacts and quantitative Xpert MTB/RIF and Xpert Ultra results. Quantification by Xpert MTB/RIF and Xpert Ultra can be used as an influential tool to prioritize contact investigation. People with PTB with negative Xpert MTB/RIF or Xpert Ultra result present a very low risk to infect household contacts.

## Supplementary Material

ofae450_Supplementary_Data

## Supplementary Data

Supplementary materials are available at *Open Forum Infectious Diseases* online. Consisting of data provided by the authors to benefit the reader, the posted materials are not copyedited and are the sole responsibility of the authors, so questions or comments should be addressed to the corresponding author.

## Note

***Potential conflicts of interest*.** All authors: No reported conflicts.
