# Supplementary material for: Usefulness of Xpert MTB/RIF and Xpert Ultra to Categorize Risk of Tuberculosis Transmission to Household Contacts
Source: Open Forum Infect Dis. 2024 Aug 6;11(8):ofae450. doi: 10.1093/ofid/ofae450 (PMC11334063; doi:10.1093/ofid/ofae450)
Supplement: ofae450_Supplementary_Data [file ofae450_supplementary_data.docx]

| **Supplementary table 1** | | | | | | | |
| --- | --- | --- | --- | --- | --- | --- | --- |
| **General Characteristics of people with extrapulmonary TB or pulmonary TB** | | | | | | | |
|  | **Overall** |  | **People with extrapulmonary TB** |  | **Included pTB cases** | **Excluded pTB cases** | **P value** |
| **Total** | 1305 (100) |  | 394 (30.2) |  | 107 (11.7) | 804 (88.3) |  |
|  |  |  |  |  |  |  |  |
| **Age, mean (SD)** | 47.6 (21.4) |  | 51.7 (21.1) |  | 47.8 (19.3) | 45.5 (21.5) | 0.296 |
| **Sex, n (%)** |  |  |  |  |  |  |  |
| Male | 763 (58.5) |  | 196 (49.7) |  | 68 (63.6) | 499 (62.1) | 0.766 |
| Female | 542 (58.5) |  | 198 (50.3) |  | 39 (36.4) | 305 (37.9) |  |
| **Country of birth, n (%)** |  |  |  |  |  |  |  |
| Spain | 463 (35.5) |  | 119 (30.2) |  | 39 (36.4) | 305 (37.9) | 0.209 |
| Outside Spain | 842 (64.5) |  | 275 (69.8) |  | 68 (63.6) | 499 (62.1) |  |
|  |  |  |  |  |  |  |  |
| **Drug use, n (%)** | 49 (3.8) |  | 5 (1.3) |  | 13 (12.1) | 31 (3.9) | <0.001* |
| **Tabacco use, n (%)** |  |  |  |  |  |  |  |
| Active smoker | 203 (15.6) |  | 36 (9.1) |  | 39 (36.4) | 128 (15.9) | <0.001* |
| Former smoker | 74 (5.7) |  | 21 (7.9) |  | 7 (6.5) | 36 (4.5) |  |
| No smoker | 874 (67.0) |  | 173 (43.9) |  | 61 (57.0) | 640 (79.6) |  |
|  |  |  |  |  |  |  |  |
| **Previous TB, n (%)** | 80 (6.1) |  | 18 (4.6) |  | 5 (5.7) | 57 (8.0) | 0.459 |
| **Immunosuppression, n (%)** | |  |  |  |  |  |  |
| DM type I | 4 (0.3) |  | 2 (0.5) |  | 0 (0.0) | 2 (0.2) | 0.597 |
| DM type II | 66 (66.0) |  | 22 (5.5) |  | 7 (6.5) | 37 (4.6) |  |
| HIV | 46 (3.5) |  | 13 (3.3) |  | 20 (18.7) | 162 (20.1) | 0.25 |
| Drug induced | 27 (2.1) |  | 8 (2.0) |  | 3 (2.8) | 16 (2.0) | 0.58 |
| Auto immune disease | 20 (1.5) |  | 6 (1.5) |  | 1 (0.9) | 13 (1.6) | 0.59 |
| Transplantation | 19 (1.5) |  | 2 (0.5) |  | 1 (0.9) | 16 (2.0) | 0.448 |
| **Symptoms, n (%)** |  |  |  |  |  |  |  |
| Respiratory | 698 (53.5) |  | 89 (22.6) |  | 87 (81.3) | 522 (64.9) | <0.001* |
| Loss of weight | 270 (20.7) |  | 53 (13.5) |  | 56 (52.3) | 161 (20.0 | <0.001* |
| sweating | 183 (14.0) |  | 24 (6.1) |  | 39 (36.4) | 120 (14.9) | <0.001* |
| Asthenia | 260 (19.9) |  | 58 (14.7) |  | 43 (40.2) | 159 (19.8) | <0.001* |
| Other | 620 47.5) |  |  |  | 24 (22.4) | 285 (35.4) | 0.008* |
|  |  |  |  |  |  |  |  |
|  |  |  |  |  |  |  |  |
|  | **Overall** |  | **Extrapulmonary TB cases** |  | **Included pTB cases** | **Excluded pTB cases** | **P value** |
| **Radiology, n (%)** |  |  |  |  |  |  |  |
| Normal | 284 (21.8) |  | 230 (58.4) |  | 1 (0.9) | 53 (6.6) | 0.009* |
| Abnormal with cavities | 225 (17.2) |  | 3 (0.8) |  | 39 (36.4) | 183 (22.8) |  |
| Abnormal without cavities | 787 (60.3) |  | 155 (39.3) |  | 67 (62.6) | 565 (70.3) |  |
| Other pathology | 6 (0.5) |  | 4 (1.0) |  | 0 (0.0) | 2 (0.2) |  |
| Not performed | 3 (0.2) |  | 2 (0.5) |  | 0 (0.0) | 1 (0.1) |  |
|  |  |  |  |  |  |  |  |
| **BCG vaccination, n (%)** | 162 (12.4) |  | 40 (10.2) |  | 17 (15.9) | 105 (13.1) | 0.396 |
| **AFB smear grading, n (%)** |  |  |  |  |  |  |  |
| 0 |  |  |  |  | 12 (11.2) | 307 (37.9) | <0.001* |
| 1 |  |  |  |  | 14 (13.1) | 28 (3.5) |  |
| 2 |  |  |  |  | 13 (12.1) | 27 (3.3) |  |
| 3 |  |  |  |  | 39 (36.4) | 52 (6.4) |  |
| 4 |  |  |  |  | 29 (27.1) | 59 (7.3) |  |
| Missing |  |  |  |  | 0 (0.0) | 337 (41.6) |  |
| **Xpert MTB quantification, n (%)** | |  |  |  |  |  |  |
| Absent |  |  |  |  | 7 (6.5) | 29 (3.3) | <0.001* |
| Very low |  |  |  |  | 6 (5.6) | 3 (0.4) |  |
| Low |  |  |  |  | 21 (19.6) | 21 (2.7) |  |
| Medium |  |  |  |  | 38 (35.5) | 14 (1.7) |  |
| High |  |  |  |  | 35 (32.7) | 33 (4.2) |  |
| Missing |  |  |  |  | 0 (0.0) | 711 (87.7) |  |
| **Infection ratio groups, n (%)** | |  |  |  |  |  |  |
| No transmission |  |  |  |  | 39 (36.4) | 152 (41.1) | 0.698 |
| 1-33% |  |  |  |  | 10 (9.3) | 41 (11.1) |  |
| 34-67% |  |  |  |  | 28 (26.2) | 82 (22.2) |  |
| 68-100% |  |  |  |  | 30 (28.0) | 95 (25.7) |  |
| Missing |  |  |  |  | 0 (0.0) | 434 (47.6) |  |
|  |  |  |  |  |  |  |  |
| *significant difference with p-value <0.05 | |  |  |  |  |  |  |

*pTB: pulmonary tuberculosis*

**Supplementary Table 2**

| **Contingency table of the distribution of the categorized infection ratios over the Xpert MTB/RIF and Xpert Ultra quantification groups** | | | | | | | | | |
| --- | --- | --- | --- | --- | --- | --- | --- | --- | --- |
| **Xpert MTB/RIF and Xpert Ultra quantification groups** | | | | | | | | | |
|  | **0** | **1** | **2** | **3** | **4** |  | **Total** |  |  |
| **No transmission** | 6 (85.7%) | 2 (33.3%) | 8 (38.1%) | 17 (44.7%) | 6 (17.1%) |  | 39 (36.4%) |  |  |
| **1-33%** | 0 (0.0%) | 1 (16.7%) | 2 (9.5%) | 6 (15.8%) | 1 (2.9%) |  | 10 (9.3%) |  |  |
| **34-67%** | 1 (14.3%) | 2 (33.3%) | 5 (23.8%) | 6 (15.8%) | 14 (40.0%) |  | 28 (26.2%) |  |  |
| **68-100%** | 0 (0.0%) | 1 (16.7%) | 6 (28.6%) | 9 (23.7%) | 14 (40.0%) |  | 30 (28.0%) |  |  |
|  |  |  |  |  |  |  |  |  |  |
| **Total** | 7 (100%) | 6 (100%) | 21 (100%) | 38 (100%) | 35 (100%) |  | 107 (100%) |  |  |
